# A five-component infection control bundle to permanently eliminate a carbapenem-resistant *Acinetobacter baumannii* spreading in an intensive care unit

**DOI:** 10.1186/s13756-021-00990-z

**Published:** 2021-08-19

**Authors:** Marianna Meschiari, José-María Lòpez-Lozano, Vincenzo Di Pilato, Carola Gimenez-Esparza, Elena Vecchi, Erica Bacca, Gabriella Orlando, Erica Franceschini, Mario Sarti, Monica Pecorari, Antonella Grottola, Claudia Venturelli, Stefano Busani, Lucia Serio, Massimo Girardis, Gian Maria Rossolini, Inge C. Gyssens, Dominique L. Monnet, Cristina Mussini

**Affiliations:** 1 Infectious Disease Clinic, Azienda Ospedaliero-Universitaria Policlinico and University of Modena and Reggio Emilia, Modena, Italy; 2 Medicine Preventive-Infection Control Team, Hospital Vega Baja, Orihuela-Alicante, Spain; 3 Department of Surgical Sciences and Integrated Diagnostics, University of Genoa, Genoa, Italy; 4 Chief. Intensive Care Unit, Hospital Vega Baja, Orihuela-Alicante, Spain; 5 Hospital Hygiene and Infection Control, Azienda Ospedaliero-Universitaria Policlinico of Modena, Modena, Italy; 6 Clinical Microbiology Laboratory, Azienda Ospedaliero-Universitaria Policlinico of Modena, Modena, Italy; 7 Laboratory of Virology and Molecular Biology, Azienda Ospedaliero-Universitaria Policlinico of Modena, Modena, Italy; 8 Anesthesia and Intensive Care Unit, Azienda Ospedaliero-Universitaria Policlinico and University of Modena and Reggio Emilia, Modena, Italy; 9 Clinical Microbiology and Virology Unit, Florence Careggi University Hospital, Florence, Italy; 10 IRCCS Fondazione Don Carlo Gnocchi, Florence, Italy; 11 Department of Experimental and Clinical Medicine, University of Florence, Florence, Italy; 12 Department of Internal Medicine and Radboud Center for Infectious Diseases, Radboud University Medical Center, 6525 GA Nijmegen, The Netherlands; 13 European Centre for Disease Prevention and Control (ECDC), Solna, Sweden

**Keywords:** Carbapenem-resistant *Acinetobacter baumannii*, Intensive care unit, Cycling radical environmental cleaning, Infection control bundle, Whole genome sequencing analysis

## Abstract

**Background:**

*Carbapenem-resistant Acinetobacter baumannii* (CRAB) infection outbreaks are difficult to control and sometimes require cohorting of CRAB-positive patients or temporary ward closure for environmental cleaning. We aimed at controlling the deadly 2018 CRAB outbreak in a 12 bed- intensive care unit (ICU) including 9 beds in a 220 m^2^ open space. We implemented a new multimodal approach without ward closure, cohorting or temporarily limiting admissions.

**Methods:**

A five-component bundle was introduced in 2018 including reinforcement of hand hygiene and sample extension of screening, application of contact precautions to all patients, enhanced environmental sampling and the one-time application of a *cycling radical environmental cleaning and disinfection* procedure of the entire ICU.

The ICU-CRAB incidence density (ID), ICU alcohol-based hand rub consumption and antibiotic use were calculated over a period of 6 years and intervention time series analysis was performed. Whole genome sequencing analysis (WGS) was done on clinical and environmental isolates in the study period.

**Results:**

From January 2013, nosocomial ICU-CRAB ID decreased from 30.4 CRAB cases per 1000 patients-days to zero cases per 1000 patients-days. Our intervention showed a significant impact (-2.9 nosocomial ICU-CRAB cases per 1000 bed-days), while no influence was observed for antibiotic and alcohol-based hand rub (AHR) consumption.

WGS demonstrated that CRAB strains were clonally related to an environmental reservoir which confirms the primary role of the environment in CRAB ICU spreading.

**Conclusion:**

A five-component bundle of continuous hand hygiene improvement, extended sampling at screening including the environment, universal contact precautions and a novel *cycling radical environmental cleaning* and disinfection procedure proved to be effective for permanently eliminating CRAB spreading within the ICU. Cohorting, admission restriction or ICU closure were avoided.

**Supplementary Information:**

The online version contains supplementary material available at 10.1186/s13756-021-00990-z.

## Background

Carbapenem-resistant *Acinetobacter baumannii* (CRAB) infections have increased over the last ten years in intensive care units (ICUs), in particular in Italy and Greece [1–3]. CRAB is difficult to eradicate from the environment due to its ability to persist on surfaces and reduced susceptibility to biocides [4, 5].

In this scenario, because of the propensity of CRAB to cause outbreaks in the healthcare setting, effective and targeted infection prevention and control (IPC) interventions are essential to stop CRAB spreading [6].

Multimodal IPC strategies appear to be highly effective for CRAB prevention and control [6, 7]. Several national and international guidelines provide evidence-based recommendations to prevent and control CRAB cross-transmission in hospital settings [8–11]. Nevertheless, controversy exists about which strategy is most pragmatic, especially in the context of limited economic and logistic resources and with regard to local epidemiology [7]. Lack of evidence is due to study designs because, in order to assess complex IPC interventions, it is often not possible to conduct a randomized controlled trial. Indeed, most intervention studies conducted in the Americas or Europe describing hospital or intensive care unit (ICU)-specific interventions were uncontrolled. The most common outcome was incidence of infection [12–16]. For CRAB, the main intervention components included alert codes, education, and environmental cleaning in addition to what are now established as hand hygiene and contact precautions. Successful control of CRAB outbreaks often required transfer of positive patients to a cohort ward [12–19] and sometimes temporary closure of the ICU [16, 20–22] Additional file 1 shows an overview of IPC components in published studies and of the present bundle ( Supplementary Material). Unfortunately, the latter practices are very difficult to implement in hospitals with only one open space ICU and limited staff and resources.

We report on the rapid and successful control of CRAB spreading in an endemic ICU, by applying an ICP bundle without cohorting CRAB-positive patients, ICU closure, or interrupting admissions.

## Methods

### Setting

**Fig. 1:**
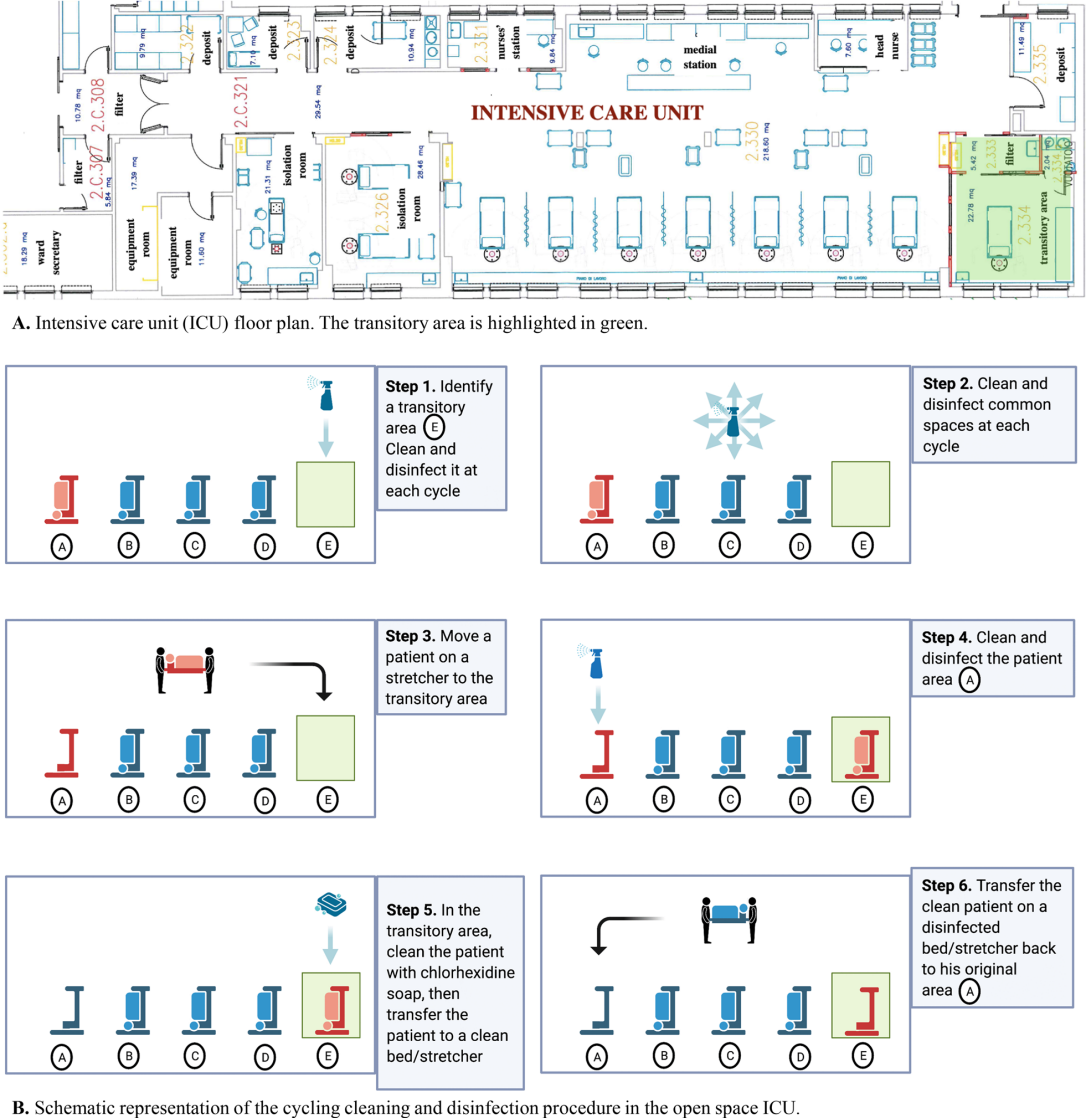
The cycling disinfection procedure. The cycling disinfection procedure consisted of terminal cleaning and disinfection of each unit with 10% sodium hypochlorite for environmental surfaces and hydrogen peroxide in wipes for all medical devices, from upper corner to opposite lower corner starting from a transitory unit to be kept free. The common areas in the ICU were cleaned and disinfected, then the colonized patient was moved from his original unit to the transitory unit in order to have the patient’s original unit disinfected. In the transitory unit, the patient’s skin was disinfected with 2% leave-on chlorhexidine disposable cloths and he was transferred to his cleaned bed. Then the patient was relocated to the cleaned and disinfected original unit and the transitory unit was subsequently cleaned and disinfected. The whole process takes on average 6 h to be completed needing the recruitment of an additional nurse crew and dedicated cleaning staff

The Azienda Ospedaliero Universitaria (AOU) Policlinico in Modena, Italy is a 700-bed tertiary care university teaching hospital. The ICU has 12 beds: three in single patient, closed, isolation rooms and nine in a 220 m^2^ open space (Fig. 1A), with a constant bed occupancy rate of 95%. The staffing proportion is one nurse for two beds.

The hospital infection control team consists of two physicians, three infection control nurses, a microbiologist, two nurses and two physicians from the ICU staff.

### Routine IPC practices and antimicrobial stewardship

Since 2011, hospital-wide rectal screening for all carbapenem-resistant Gram-negative bacteria (CR- GNB) is performed at admission and repeated weekly. An active surveillance system involving the microbiology laboratory and infection control staff allows to promptly identify all patients colonized or infected with CR-GNB. CR-GNB-colonized patients are cared for with contact precautions, using gowns and gloves for any patient contact. As from 2012, a multimodal project of hand hygiene according to WHO recommendations[23] was implemented. An antimicrobial stewardship program, in addition to standard consultations, has started since September 2014, with audit and immediate feedback three times per week performed by an infectious disease specialist, and a restricted formulary for carbapenems, fluoroquinolones, colistin, and tigecycline.

Routine twice daily standard cleaning of the single patient units (i.e. isolation rooms and open space areas) with 10% sodium hypochlorite for environmental surfaces and hydrogen peroxide in wipes for all medical devices.

CRAB was endemic in the ICU and the hospital. Between February and March 2018, 5 CRAB-infected patients died in the ICU (Table 1), and this was the reason for intensifying ICP measures. This period is termed “CRAB outbreak”.

**Table 1:** Characteristics of patients and CRAB isolates, February–March 2018

Patient Number	Age/sex	Date of ICU admission	Date of discharge/death	Outcome	Isolate number	Date of isolation	Type of sample	Pattern of acquisition	Type of infection	WGS code
P1	55/F	25/02	12/04	Death	A1.1	26-2-18	BAL	Infection	VAP+ BSI	Ab284
					A1.2	27-2-18	Blood	Infection		Ab278
P2	58/F	22/02	28/02	Death	A2.1	28-2-18	Blood	Infection	BSI	Ab282
P3	9/F	04/03	13/03	Discharge	A3.1	8-3-18	Pharynx	Colonization	–	Ab275
					A3.2	8-3-18	Axillary	Colonization	–	Ab273
P4	52/F	04/03	23/03	Discharge	A4.1	14-3-18	Urine	Colonization	–	Ab287
P5	73/M	27/02	27/03	Death	A5.1	15-3-18	BAL	Infection	HAP	Ab286
P6	66/F	11/03	24/03	Death	A6.1	22-3-18	Blood	Infection	BSI	Ab276
P7	83/F	26/03	29/03	Death	A7.1	28-3-18	Blood	Infection	BSI	Ab277

Coding of isolates according to WGS

Colonization with CRAB was defined as the detection of this pathogen in swabs without any evidence of clinical disease. BAL: Broncho-alveolar lavage; BSI: Bloodstream infection; CRAB: carbapenem-resistant *A. baumannii*; HAP: Hospital acquired pneumonia; VAP: Ventilator associated pneumonia; WGS: whole genome analysis

### Intervention

The intervention in the ICU was a five-component bundle implemented over time during the outbreak.

- A long-term component of the bundle started from 28th February 2018 and was continued thereafter:

1. Proactive reinforcement of all routine IPC practices among healthcare workers:
  - improving hand hygiene compliance with 100 direct observations of the “5 moments” opportunities performed by IPC nurses according to the WHO [23] guidelines followed by individualized verbal feedback
  - creating an “improvement group” with medical and nursing staff to analyse critical issues regarding hand hygiene compliance
  - monitoring compliance with contact precautions performed by IPC nurses using two specific checklists
  - meetings with radiology and transport personnel to reinforce compliance with IPC measures
2. Extended screening. All patients with an expected length of stay on the ICU for > 24 h were screened for the carriage of CRAB. This was done by collecting the following samples at admission and weekly thereafter: swabs from the axilla, the groin, the trachea in addition to the rectum. Screening samples were performed using selective MacConkey agar plates (bioMérieux Firenze, Italia) with meropenem (10 µg) disks.
3. Contact precaution measures for all patients until discharge, independently of CRAB status (Personal protective equipment for each single patient unit included wearing single-use gloves and gowns before entering, changing the gloves according to WHO 5 moments for hand hygiene).
4. Environmental sampling using pre-moistened sterile gauze pads; the procedure previously recommended by Corbella et al. [24] was introduced in order to increase sensitivity. All ICU surfaces were vigorously rubbed by means of moistened sterile gauze pads in a screw-cap container with 10 mL of brain heart infusion medium (BHI). After 24 h of incubation at 37 °C in BHI, gauzes were sampled into MacConkey agar plates and incubated aerobically at 37 °C for 48 h.

- A short-term component of the bundle that was applied only once:

5. *Cycling radical cleaning and disinfection* of all rooms, areas and patients, (Fig. 1B), detailed below.

In summary, among the above-mentioned intervention strategies, components 4 and 5 were de novo introduced, components 2 and 3 were intensified and revised (e. g. universal *versus* target contact precautions; multiple sites active screening *versus* rectal screening only), component 1 was reinforced as it was applied intermittently in the previous years.

To further investigate the outbreak, whole genome sequencing (WGS) analysis was performed on sixteen CRAB isolates, including all seven environmental and a selection of nine clinical isolates. Clinical isolates for genotyping were selected giving priority to diagnostic samples from sterile body sites, surveillance samples were included only in the absence of clinical ones in order to collect at least one sample per patient. Microbiological analysis and whole genome analysis sequencing are detailed in Additional file 2.

### Cycling radical cleaning procedure

A novel radical cleaning procedure was performed on April 12nd (Fig. 1B). This method was applied only once and consisted of terminal cleaning and disinfection of each unit with 10% sodium hypochlorite for environmental surfaces and hydrogen peroxide in wipes for all medical devices, from upper corner to opposite lower corner starting from a *transitory* unit to be kept free.

The disinfectants were allowed to dry completely before re-using the surface. The common areas in the ICU were disinfected, then the colonized patient was moved from his *original* unit to the *transitory* unit in order to have the patient’s *original* unit disinfected. In the *transitory* unit, the patient’s skin was disinfected with 2% leave-on chlorhexidine disposable cloths and he was transferred to his cleaned bed. Then the patient was relocated to the disinfected *original* unit and the *transitory* unit was subsequently disinfected. The whole process takes in average 6 h to be completed needing the recruitment of an additional nurse shift and dedicated cleaning staff of 2 people.

Cleaned surfaces were checked by infection control nurses using fluorescein spray with an UV torch. Fluorescent spots indicated that the surface had not been cleaned effectively, and disinfection was repeated. Thanks to this simple procedure, it was possible to easily check if the surfaces were truly cleaned, especially in hard-to-reach areas. It also helped to establish whether there was a need for staff re-training or a change in cleaning practices.

### Data collection and definitions

For each CRAB infected or colonized patient, demographic and clinical characteristics were collected from the electronic medical records.

Hospital acquired infection (HAI) was defined according to the criteria used by the Centres for Disease Control and Prevention [25]. Colonization with CRAB was defined as the detection of this pathogen in swabs without any evidence of clinical disease. Episodes of colonization or infection were considered ICU-acquired if they were not present at the time of admission and were acquired after 48 h from ICU admission (nosocomial ICU-CRAB). Conversely, CRAB events were defined as imported to ICU if they were detected on ICU-admission by screening or appeared within 48 h from hospital admission (imported-ICU-CRAB). The monthly Incidence Density (ID) of nosocomial ICU-CRAB and imported ICU-CRAB was also calculated by dividing the number of cases (including both colonization and infections) by the total number of days of ICU stay of all patients at risk and expressed per 1000 patient-days from January 2013 to December 2019.

Compliance with hand hygiene was assessed through ICU monthly consumption of alcohol-based hand rubs (AHR) expressed as litres per 1000 bed-days. Carbapenem and fluoroquinolone consumption was calculated using ICU monthly consumption data, expressed as the number of Defined Daily Doses (DDDs) per 1000 bed-days.

### Statistical analysis

To detect a possible change in the CRAB ID trends in ICU after the two kinds of interventions (long-term and short-term as defined in the Intervention section), an Intervention Time Series Analysis was conducted using monthly data (a total of 82 observations) [26–29]. First, a graphic exploration of the series was performed. Line plots at monthly time series were produced for nosocomial ICU-CRAB ID, for imported ICU-CRAB ID, for the use of each antimicrobial drug class and for AHR use to examine their evolution over time and to compare their respective impact.

Once the basic characteristics (i.e., autocorrelation, seasonality, and general and post-intervention trends) of each of the above-mentioned time series were established, a multivariate analysis was performed to quantify the relationships between use of the mentioned antimicrobial classes, AHR use, imported ICU-CRAB ID and Intervention over nosocomial ICU-CRAB ID by using dynamic time-series modelling techniques. For this purpose, a Linear Transfer Function (LTF) model was built according to the identification method proposed by Pankratz [26] and Lon-Mo Liu [27]. In the LTF model we included a binary term (coded as 0 before the long-term intervention and 1 afterward), as well as an additional binary term for the short-term intervention (coded as 1 for April 2018 only and as 0 thereafter), to estimate the impact of the intervention controlling for the rest of mentioned variables. We followed a backward modelling strategy, initially considering all series as possible explaining series, including ICU-CRAB ID (except trends related) lagged from 1 to 4 periods and eliminating the lags that did not show a significant relationship (p $\le$ 0.05). Data were analysed with SCA release 8.1 [Scientific Computer Associates, Chicago (IL), www.scausa.com].

## Results

### Outbreak description

The index patient (P1) came from Greece and was admitted to the ICU on February 25th while waiting for a liver retransplant after receiving a first transplant in Athens in January 2018. CRAB was isolated from the surveillance rectal swab taken on admission and on February 27th she developed a CRAB bloodstream infection (BSI). From the end of February to mid-March 2018, six other ICU patients were found positive for CRAB: four were infected (P2, P5, P6 and P7) and two colonized (P3 and P4). All patient surveillance samples collected at ICU admission were negative for CRAB. Characteristics of the patients and CRAB isolates collected during the outbreak are shown in Table 1.

Patient history, clinical course and characteristics are fully described in Additional file 2 and in Additional file 1: Table 1, within the same Additional file 2. Antimicrobial susceptibility testing of the CRAB clinical isolates is shown in 1: Table 3 (Additional file 2)*.*

### Environmental sampling during the outbreak

To compare the sensitivity of environmental sample collection methods, two procedures were performed in parallel: standard technique through cotton applicator swabs and the newly introduced BHI moistened sterile gauze technique.

After the death of P2, three out of 10 environmental samples collected with sterile BHI moistened gauze, all bordering on the P1 and P2 area, were positive for CRAB, including: bedside table handle in the P1 area (isolate code Ab274), linen counter handle in front of the P2 area (Ab280) and emergency joint cart located in the open space (Ab281). Conversely, no bacterial growth was observed using standard cotton swabs of the same surfaces. We can assume that emergency joint cart (Ab281) promoted the CRAB cross-transmission between P2 and the other three patients (P3, P4, P5).

After the death of P1, standard terminal cleaning of her isolation room using 10% sodium hypochlorite was performed and 10 environmental samples were collected according to the CDC Environmental Checklist for Monitoring Terminal Cleaning, paying particular attention to high-touch surfaces. Despite the cleaning, four out of ten environmental samples collected by the moistened gauze method of P1’s room yielded CRAB, including multi-monitor controls (Ab272), P1 IV pole (grab area) (Ab283), medical equipment cart/medication cart (Ab279), P1 bed (Ab285). As before, environmental cultures obtained using standard technique yielded negative results. Comparing the two procedures, both performed in all sampling, BHI moistened sterile gauze technique was a more sensitive method for CRAB detection (40% positives vs 0%; P < 0.05), and subsequently replaced the standard technique. After applying *cycling radical cleaning and disinfection* and using fluorescein spray for monitoring the effectiveness of terminal cleaning, no sample obtained with the moistened gauze technique was positive for CRAB in the whole ICU.

### Outbreak profiling by WGS analysis

**Fig. 2:**
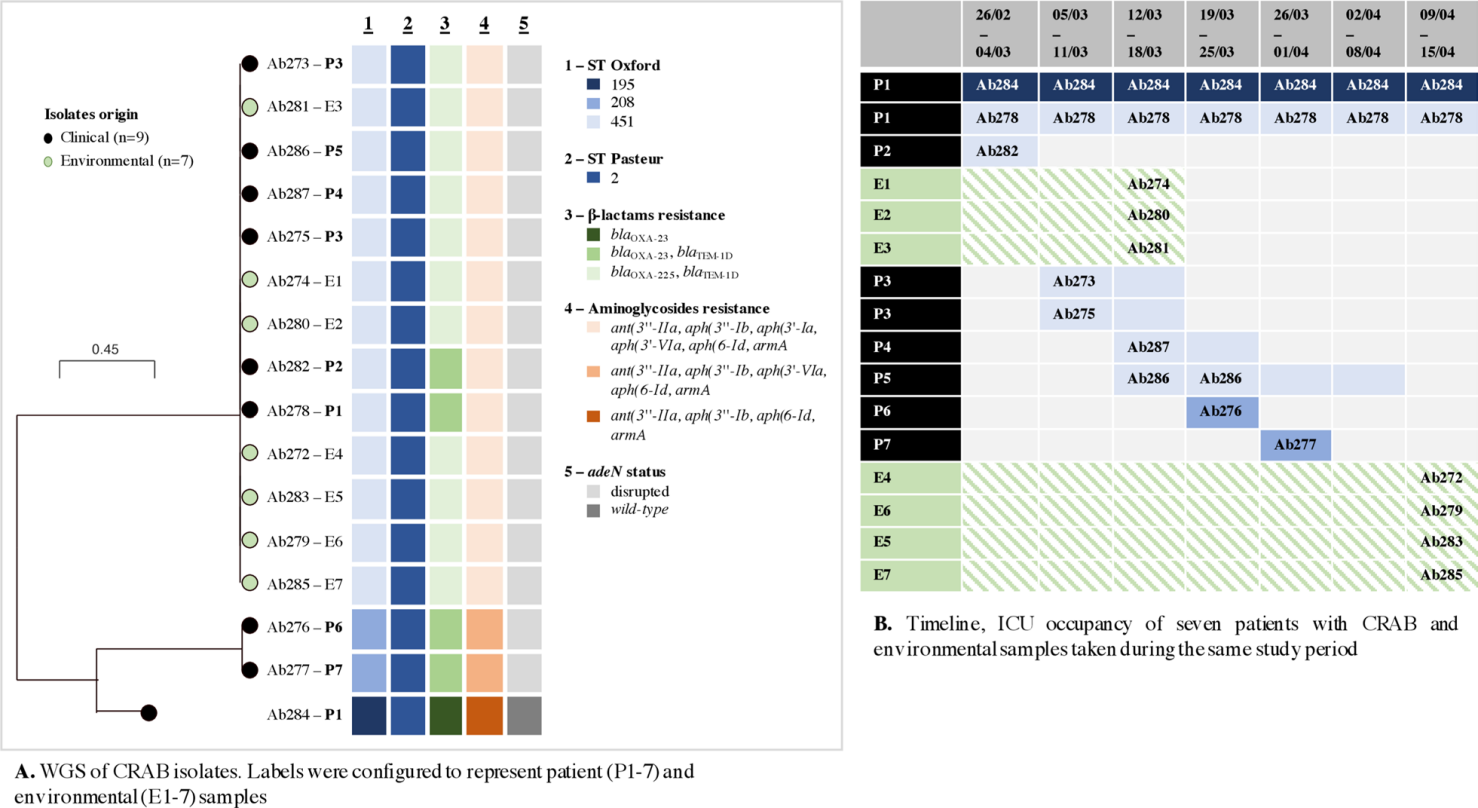
Outbreak profiling with WGS analysis and inference of the most likely transmission events. WGS: whole genome sequencing; CRAB: carbapenem-resistant *A. baumannii*; ICU: intensive care unit. Patients’ samples refer to: P1: blood (25/2, 27/2, 13/3,20/3, 22/3, 3/4, 10/4), bronchial aspirate (26/2,4/3,10/4), bile (25/2), peritoneal fluid (25/2, 10/4), urine (26/2), rectal swab (26/2,4/3,13/3,17/3,22/3,4/4), skin (26/2,4/3,13/3,17/3,22/3,4/4); P2: blood (28/2), urine (28/2), skin (28/2), rectal swab (28/2); P3: skin (8/3); P4: urine (14/3,18/3), central venous catheter insertion tip (14/3), skin (14/3), rectal swab (14/3); P5: bronchial aspirate (15/3, 21/3), rectal swab (16/3, 20/3), skin (16/3, 20/3); P6: blood (22/3), bronchial aspirate (22/3), decubitus ulcers (22/3), skin (22/3), rectal swab (22/3); P7: blood (28/3), bronchial aspirate (27/3), skin (27/3), rectal swab (27/3). Environmental samples refer to: Ab274: bedside table handle in the P1 area; Ab280: linen counter handle in front of the P2 area; Ab281: emergency joint cart located in open bay space; Ab272: multi-monitor controls; Ab279: medical equipment cart/ medication cart; Ab283: P1 IV pole (grab area); Ab285: P1 bed

In silico determination of MLST according to the Pasteur scheme revealed that all 16 isolates belonged to sequence type (ST) 2, that is part of the international clone II lineage. Conversely, determination of MLST according to the Oxford scheme revealed the presence of three distinct clones, of which ST451 was the most prevalent (n = 13) over ST208 (n = 2) and the singleton ST195 (Fig. 2A).

Evaluation of core genome single nucleotide polymorphisms (SNPs) corroborated the presence of multiple CRAB clones during the outbreak. Indeed, the genetic diversity observed between ST451 and ST208 (SNPs range: 1148–1151; mean\median: 1149) or ST195 (SNPs range: 1050–1053; mean\median: 1051), as well as between ST208 and ST195 (809 SNPs), was significantly higher than that observed within each clone. In fact, only few SNPs were identified within ST451 isolates (SNPs range: 0–7; mean\median: 2) while no SNPs were detected within the two ST208 isolates.

Screening for acquired resistance determinants revealed that, except for the single ST195 isolate not producing TEM-1D, all isolates carried genes coding for TEM-1D and OXA-23 or OXA-225 (OXA-23-like) -type carbapenemases; additionally, a clone-specific content of genes coding for aminoglycoside resistance was observed (Fig. 2A).

Screening for virulence traits revealed the presence of an insertional inactivation of the *adeN* gene, coding for a negative regulator of the AdeIJK efflux system. The inactivation of adeN was detected in all sequenced isolates except ST195, belonged to respiratory sample collected from P1 (Ab284).

### Inference of the most likely transmission events

Based on WGS and patient data, the following epidemiological scenario was hypothesized. P1, arriving from Greece, was apparently colonised by two different CRAB clones of ST195 (Ab284) and ST451 (Ab278), respectively, of which the latter caused the bloodstream infection (BSI) episode observed at admission. ST451 subsequently spread in the ward, with contamination of environmental surfaces and cross-transmission to other patients (P2, P3, P4 and P5) who experienced either colonization or infection. Indeed, isolation of an ST451 CRAB (Ab281) highly related to Ab278 from the emergency joint cart three days after identification of the index case, strongly suggests that there was an early contamination of the ICU environment with this strain, which apparently persisted until mid-April, as demonstrated by the positivity for closely related ST451 CRAB (Ab272, Ab283, Ab279, Ab285) of environmental samples collected at that time (Fig. 2B). On the other hand, despite concomitant ICU stay, P6 and P7 were infected with a different clone belonging to ST208, showing a different resistome profile compared to that of ST451, which was apparently derived from an additional CRAB introduction within the ICU setting (Fig. 2B).

### ICU-CRAB epidemiology: results from the intervention time series analysis

**Fig. 3:**
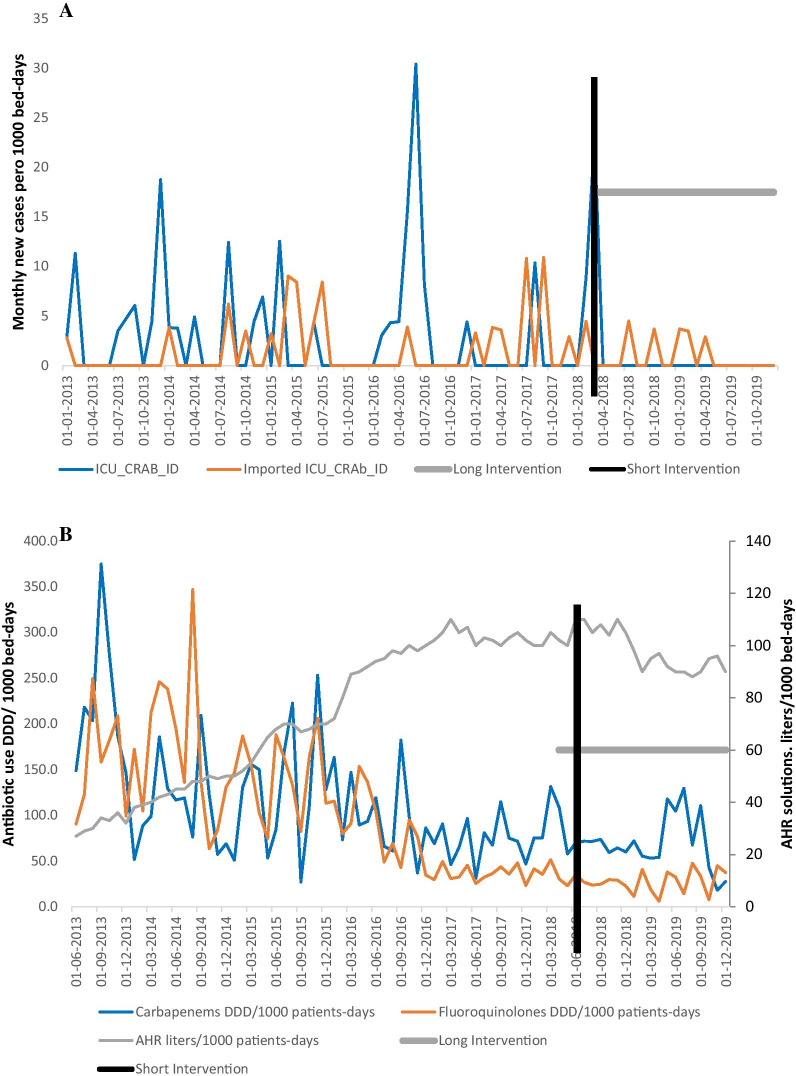
Graphic representation of monthly Incidence Density of new nosocomial and imported cases of ICU CRAB (A); antibiotic and AHR consumptions (B), January 2013- January 2019. Thick grey line: long intervention. Black line: short intervention. **A** Blue line: nosocomial ICU-CRAB ID. **A** Orange line: imported-ICU-CRAB ID. **B** Blue line: Carbapenems DDD/1000 patients-days. **B** Orange line: Fluoroquinolones DDD/1000 patients-days. **B** Thin grey line: alcohol-based hand rub (AHR) consumption in litres /1000 patients-days. The multimodal intervention started on February 28th, 2018

Figure 3A shows the monthly CRAB ID trend distinguishing between nosocomial ICU and imported ICU isolates. From January 2013, nosocomial ICU-CRAB ID varied considerably, peaking at 30.4 and 25 CRAB cases per 1,000 patients-days in January 2016 and January 2018, respectively. Following the intervention, nosocomial ICU-CRAB ID achieved zero cases per 1000 patients-days: no new nosocomial ICU-CRAB case was identified from April 2018 onwards, after the cycling cleaning procedure was performed, apart from sporadic imported CRAB cases in patients admitted from community or from other hospitals.

From 2013 to 2019, ICU AHR use increased progressively from 30 to 100 L per 1000 patient-days. Total Antibiotic use decreased from 209 to 171 DDD/1000 patients-days (data not shown), while carbapenems and fluoroquinolone use decreased from 60 to 10 and from 50 to 8 DDD/1000 patients-days, respectively (Fig. 3B).

After identifying an Intervention Time Series Analysis model with all significant parameters, the short-term Intervention showed a significant impact (-7.2 ICU cases per 1000 bed-days) while the impact of the long-term intervention was also significant: -2.22 cases per 1000 bed-days (Table 2). The series imported ICU-CRAB ID showed a positive impact on nosocomial ICU-CRAB ID lagged by one month (each new case of imported ICU CRAB will select 0.67 new ICU CRAB cases). There was also a significant influence of past abrupt changes (t-2 and t-5 months) and the previous month of the same ICU-CRAB ID. Conversely, we did not observe a statistically significant effect of antibiotic and AHR use on ICU-CRAB ID, probably because the AHR use had already increased (2015/2016) and antimicrobial use had already decreased (2016/2017) prior to the intervention (Fig. 3B).

**Table 2:** Change in the ICU-CRAB ID: results from the Intervention Time Series Analysis model

Explanatory variables	Lag (months)	Coefficents	Standard error	T-ratio	*p* value
Constant	0	2.1900	0.2112	10.37	< 0.001
Short-term Intervention	0	− 7.2215	3.4530	− 2.09	< 0.05
Long-term Intervention	0	− 2.2161	0.6353	− 3.49	< 0.01
Imported CRAB	1	0.7156	0.1534	4.67	< 0.01
ICU-CRAB (MA)	2	0.4791	0.0940	5.09	< 0.01
ICU-CRAB (MA)	5	0.6079	0.0877	6.93	< 0.001
ICU-CRAB (AR)	1	0.4149	0.097	4.26	< 0.01

R^2^: 0.352, Effective Number of observations: 82

Interpretation of coefficients:

Every change by one unity of an explaining series, implies a change of (the value of the coefficient) lagged by calculated lags (in month) in the dependent series (± t*SE)

The short-term intervention coefficient means that in the period postintervention a diminution of -7.2 (± 1.96*3.3452*0.69) new cases of nosocomial ICU CRAB/1000 bed-days was observed

The long-term intervention coefficient means that in the period postintervention a diminution of 2.2 (± 1.96*0.63) new cases of nosocomial ICU CRAB/1000 bed-days was observed

Imported CRAB term means that for every new imported ICU case, 0.72 new ICU cases occurred one month later

ICU CRAB term indicates the impact of past values of the own dependent series:

- AR: Autoregressive term, reflecting impact of previous CRAB-ID (past inertia lagged by 1 month)

- MA: Moving Average term (past abrupt changes lagged by 2 and 5 months)

ICU-CRAB ID: nosocomial colonization or infection by carbapenem-resistant *A. baumannii* Incidence Density

## Discussion

To finally control nosocomial CRAB in our ICU, we successfully applied a “five-component bundle”, which consisted of reinforcement of previously recommended measures [7, 10, 11, 17] and innovative actions. The novel procedure *Cycling radical cleaning and disinfection* allowed us to avoid ICU closure and limiting admissions. Moreover, contrary to previously reported experiences [20–22], we did not need cohorting of patients. This intervention, although being labor-intensive, was applicable in our open space ICU, a type of ICU which is the most affected by nosocomial epidemics [28]. Our experience can help hospitals with single ICUs facing similar outbreaks and endemicity.

Since in many hospitals it is unthinkable to close the hospital’s only ICU, this procedure should be considered in such settings as it can be completed on average in 6 h with an additional crew of trained staff. It also avoids restricting ICU admissions. Furthermore, patient cohorting is hard to implement in an open space ICU and has a high probability of failure because of low compliance and difficulties in full cohorting of all medical staff, cleaning staff and consultants. Nurse cohorting, which often requires an additional nurse shift, is even more difficult.

To date, no published interventions avoided at least temporary closure or cohorting of colonized patients to limit CRAB spread in an ICU (Additional file 1). Moreover, considering that ward closures and temporary limiting admissions contributed to the largest part of the total costs for outbreak control [29], these findings could be valuable.

The efficacy of a multimodal approach on CRAB outbreak control has been widely underlined in several guidelines and single center experiences [8–20]. However, our intervention study added a new element in the control of this hard-to-treat pathogen and clarified the transmission dynamics of CRAB, an issue still under investigation [30]. Environmental contamination appeared to represent the most frequent source of CRAB cross-transmission in ICU.

By using intervention time-series analysis, we demonstrated that an ICP bundle including enhanced environmental cleaning had a decisive impact on nosocomial CRAB ICU incidence density against a background of stable AHR and antibiotic use. The short-term Intervention was three times more effective than the long-term one, because it eliminated the chronic load of environmental bacterial contamination.

Valencia-Martìn et al. underlined that the major limitation of the design of a multimodal program is that it usually precludes the understanding of which is the most effective strategy to eradicate CRAB from ICUs, given that all strategies are applied simultaneously [16]. Indeed, we cannot determine the effect of each individual component of the bundle. Another relevant observation is that the colonization pressure, represented in the time series by the Non-ICU CRAB ID, works as an amplifier of nosocomial cases. This underlines that screening strategies and immediate implementation of contact isolation of CRAB carriers are key elements to prevent CRAB nosocomial transmission and subsequent infections.

Additionally, in line with previous evidence [5], we demonstrated that standard cleaning with self-monitoring is insufficient to control the CRAB environmental spread. There are several promising emerging technologies for environmental cleaning and disinfection, but they are expensive, poorly tolerable and require a substantial amount of time before room release for any new patient [14, 20]. As an example, the “No-touch” cleaning methods, such as UV and pressurized hydrogen peroxide, are undoubtedly promising but require the room to be tightly closed and unoccupied. Due to these limitations, we used 10% sodium hypochlorite and hydrogen peroxide whilst focusing on increasing compliance through the routine use of the CDC Environmental Checklist for Monitoring Terminal Cleaning [31]. We strongly believe that mechanical removal of biofilm may be more relevant than the type of disinfectant used with regard to *Acinetobacter* [21]. In order to reinforce compliance, we decided to keep on monitoring the completeness of the cycling radical environmental cleaning and disinfection by using fluorescein spray, not only to confirm that all surfaces were cleaned, but also to raise awareness and educate cleaning staff about terminal cleaning. This method has the advantages of being fast, easy to use and cost-effective, meeting the need for an external validation of room cleaning, which is mandatory in such endemic setting.

With regard to CRAB active screening strategies, an universal consensus has not been reached yet [10]. The Task force on management and prevention of *Acinetobacter baumannii* infections in the ICU has recommended weekly rectal, pharyngeal and tracheal swabs [9]. A recently published program by Valencia-Martìn et al. found a sensitivity of 96% combining rectal and pharyngeal swabs compared to 78% of rectal swab only [16]. We chose to implement active screening with rectal and skin swabs, but also respiratory samples such as endotracheal aspirates. The best performance was obtained by skin samples (100%), followed by the rectal samples (86%). As the results of CRAB screening are not immediately available, we suggest applying contact precautions to all ICU patients until outbreak termination.

Environmental sampling of *A. baumannii* through standard swabbing has proven to be sub-optimal [13], with sensitivity rates ranging from 0 to 18%, according to several factors such as the extension of the outbreak and the sampling technique used. By using BHI pre-moistened sterile gauze pads, more than 50% of our environmental samples were positive for CRAB. On the basis of this gain in sensitivity, the BHI pre-moistened method became the method of choice for CRAB environmental detection in the whole hospital.

The CRAB isolates obtained from the environmental sampling represented a precious resource to investigate transmission in the ICU and to understand how the outbreak evolved. Indeed, sequencing data strongly suggest that transmission events not fully explained by patients’ overlapping stays could primarily result from contamination of the environment, leading to a more complicated transmission network. In this context, WGS played a fundamental role in distinguishing highly related clones accounting for different introduction events, and in identifying potential environmental reservoirs close to the patient leading to perpetuation over time. The high discriminatory power provided by WGS has already been proven by other authors [32, 33], even if these studies were not able to demonstrate a link with an environmental source. Noteworthy, WGS could provide a key contribution to identify specific virulence-associated genetic variants. Indeed, a non-functional adeN was recently associated with a drastic increase in the virulence potential and with a hyper invasiveness in in vivo models using *G. mellonella* and the A549 cell line, respectively (33). During the outbreak, only two patients who acquired a CRAB clone with inactivation of adeN survived, probably due to their younger age and less immune-compromised status. Such increase in virulence may have contributed to the high mortality rates of our outbreak, which unfortunately did not differ from that reported in literature [34–36], despite the compassionate use of cefiderocol [37] in two patients.

Finally, our results should be interpreted with caution considering the main limitations: first, this is a single center study whose conclusions are not directly transferable to other facilities; second, it has been conducted in a 12 bed ICU with only 3 isolation rooms and this could have facilitated the cross-transmission; third, the low use of antibiotics and the high consumption of AHR, as well as their lack of abrupt changes during the Intervention and post-intervention periods, may have hampered observing the logical and expected impact of these factors on the resistance; fourth, this analysis does not allow to compare the impact of antibiotics and AHR use vs the effect of the ICP bundle, because we were only able to measure the impact of the two kinds of outbreak interventions.

## Conclusions

The application of this five-component bundle directed not only towards the patient (as source), but more importantly, aiming at eliminating environmental contamination, was dramatically effective in eliminating nosocomial CRAB from the ICU. In addition to more acknowledged strategies, a novel procedure *cycling radical cleaning and disinfection* was used. Advanced genotyping methods, in particular WGS, proved to be a valuable tool for identification of the sustained reservoirs.

Our successful real-life experience could help intensive care clinicians facing the huge challenge of CRAB control in ICUs with limited resources. The main advantages of our bundle are its low cost, applicability in open-space areas without cohorting, limiting admissions or ICU closures.

## Supplementary Information

**Additional file 1**. The comparison of different infection control interventions reported in literature for the management of CRAB outbreaks in ICUs.

**Additional file 2**. Microbiological analysis and whole genome sequencing analysis are illustrated technically. Patients’ history, clinical course and characteristics are fully described in text and in Supplementary Table 2. Antimicrobial susceptibility testing of the CRAB clinical isolates is shown in Supplementary Table 3.

## Data Availability

The datasets used during the current study are available from the corresponding author on reasonable request.

## Abbreviations

AHR: Alcohol-based hand rub
BSI: Bloodstream infection
CR-GNB: Carbapenem-resistant Gram-negative bacteria
CRAB: Carbapenem-resistant *Acinetobacter baumannii*
DDDs: Defined Daily Doses
HAI: Hospital acquired infection
ICU-CRAB: Nosocomial colonization or infection by carbapenem-resistant *Acinetobacter baumannii*
ICU-CRAB ID: Nosocomial colonization or infection by carbapenem-resistant *Acinetobacter baumannii* Incidence Density
ICU: Intensive care unit
ID: Incidence density expressed per 1000 patient-days
IPC: Infection prevention and control
Nosocomial ICU-CRAB: Episodes of colonization or infection were considered ICU-acquired if they were not present at the time of admission and were acquired after 48 h from ICU admission. Imported-ICU-CRAB: Episodes of colonization or infection were considered imported to ICU if they were detected on ICU-admission by screening or appeared within 48 h from hospital admission
SNPs: Single nucleotide polymorphisms
ST: Sequence type
WGS: Whole genome sequencing analysis

## References

[1] Munoz-Price LS, Weinstein RA (2008). Acinetobacter infection. N Engl J Med.

[2] Cristina ML, Spagnolo AM, Ottria G, Sartini M, Orlando P, Perdelli F (2011). Spread of multidrug carbapenem-resistant Acinetobacter baumannii in different wards of an Italian hospital. Am J Infect Control.

[3] Cassini A, Högberg LD, Plachouras D, Quattrocchi A, Hoxha A, Simonsen GS (2019). Attributable deaths and disability-adjusted life-years caused by infections with antibiotic-resistant bacteria in the EU and the European Economic Area in 2015: a population-level modelling analysis. Lancet Infect Dis.

[4] Marchaim D, Navon-Venezia S, Schwartz D, Tarabeia J, Fefer I, Schwaber MJ (2007). Surveillance cultures and duration of carriage of multidrug-resistant Acinetobacter baumannii. J Clin Microbiol.

[5] Lerner AO, Abu-Hanna J, Carmeli Y, Schechner V (2020). Environmental contamination by carbapenem-resistant Acinetobacter baumannii: the effects of room type and cleaning methods. Infect Control Hosp Epidemiol.

[6] Wieland K, Chhatwal P, Vonberg R-P (2018). Nosocomial outbreaks caused by Acinetobacter baumannii and Pseudomonas aeruginosa: results of a systematic review. Am J Infect Control.

[7] Tomczyk S, Zanichelli V, Grayson ML, Twyman A, Abbas M, Pires D (2019). Control of carbapenem-resistant enterobacteriaceae, acinetobacter baumannii, and pseudomonas aeruginosa in healthcare facilities: a systematic review and reanalysis of quasi-experimental studies. Clin Infect Dis.

[8] Guide to the Elimination of Multidrug-resistant Acinetobacter baumannii Transmission in Healthcare Settings (2010) [Internet]. APIC. [cited 2020 Sep 24]. Available from: https://apic.org/guide-to-the-elimination-of-multidrug-resistant-acinetobacter-baumannii-transmission-in-healthcare-settings-2010/

[9] Garnacho-Montero J, Dimopoulos G, Poulakou G, Akova M, Cisneros JM, De Waele J (2015). Task force on management and prevention of Acinetobacter baumannii infections in the ICU. Intensive Care Med.

[10] Tacconelli E, Cataldo MA, Dancer SJ, De Angelis G, Falcone M, Frank U (2014). ESCMID guidelines for the management of the infection control measures to reduce transmission of multidrug-resistant Gram-negative bacteria in hospitalized patients. Clin Microbiol Infect.

[11] WHO | Guidelines for the prevention and control of carbapenem-resistant Enterobacteriaceae, *Acinetobacter baumannii* and *Pseudomonas aeruginosa* in health care facilities [Internet]. WHO. World Health Organization; [cited 2020 Sep 24]. http://www.who.int/infection-prevention/publications/guidelines-cre/en/29630191

[12] Munoz-Price LS, Carling P, Cleary T, Fajardo-Aquino Y, DePascale D, Jimenez A (2014). Control of a two-decade endemic situation with carbapenem-resistant Acinetobacter baumannii: electronic dissemination of a bundle of interventions. Am J Infect Control.

[13] Enfield KB, Huq NN, Gosseling MF, Low DJ, Hazen KC, Toney DM (2014). Control of simultaneous outbreaks of carbapenemase-producing enterobacteriaceae and extensively drug-resistant Acinetobacter baumannii infection in an intensive care unit using interventions promoted in the Centers for Disease Control and Prevention 2012 carbapenemase-resistant Enterobacteriaceae Toolkit. Infect Control Hosp Epidemiol.

[14] Gray AP, Allard R, Paré R, Tannenbaum T, Lefebvre B, Lévesque S (2016). Management of a hospital outbreak of extensively drug-resistant Acinetobacter baumannii using a multimodal intervention including daily chlorhexidine baths. J Hosp Infect.

[15] Karampatakis T, Tsergouli K, Iosifidis E, Antachopoulos C, Karapanagiotou A, Karyoti A (2018). Impact of active surveillance and infection control measures on carbapenem-resistant Gram-negative bacterial colonization and infections in intensive care. J Hosp Infect.

[16] Valencia-Martín R, Gonzalez-Galan V, Alvarez-Marín R, Cazalla-Foncueva AM, Aldabó T, Gil-Navarro MV (2019). A multimodal intervention program to control a long-term Acinetobacter baumannii endemic in a tertiary care hospital. Antimicrob Resist Infect Control.

[17] Cho O-H, Bak MH, Baek EH, Park K-H, Kim S, Bae I-G (2014). Successful control of carbapenem-resistant Acinetobacter baumannii in a Korean university hospital: a 6-year perspective. Am J Infect Control.

[18] Chung YK, Kim J-S, Lee SS, Lee J-A, Kim H-S, Shin K-S (2015). Effect of daily chlorhexidine bathing on acquisition of carbapenem-resistant Acinetobacter baumannii (CRAB) in the medical intensive care unit with CRAB endemicity. Am J Infect Control.

[19] Zhao Y, Hu K, Zhang J, Guo Y, Fan X, Wang Y (2019). Outbreak of carbapenem-resistant Acinetobacter baumannii carrying the carbapenemase OXA-23 in ICU of the eastern Heilongjiang Province. China BMC Infectious Diseases.

[20] Molter G, Seifert H, Mandraka F, Kasper G, Weidmann B, Hornei B (2016). Outbreak of carbapenem-resistant Acinetobacter baumannii in the intensive care unit: a multi-level strategic management approach. J Hosp Infect.

[21] Ben-Chetrit E, Wiener-Well Y, Lesho E, Kopuit P, Broyer C, Bier L, et al. An intervention to control an ICU outbreak of carbapenem-resistant Acinetobacter baumannii: long-term impact for the ICU and hospital. Crit Care [Internet]. 2018 [cited 2020 Sep 24];22. https://www.ncbi.nlm.nih.gov/pmc/articles/PMC6249923/10.1186/s13054-018-2247-y PMC6249923 30463589

[22] Metan G, Zarakolu P, Otlu B, Tekin İ, Aytaç H, Bölek EÇ (2020). Emergence of colistin and carbapenem-resistant Acinetobacter calcoaceticus-Acinetobacter baumannii (CCR-Acb) complex in a neurological intensive care unit followed by successful control of the outbreak. J Infect Public Health.

[23] World Health Organization. WHO guidelines on hand hygiene in healthcare. 2009. http://whqlibdoc.who.int/publications/2009/9789241597906_eng.pdf

[24] Corbella X, Pujol M, Argerich MJ, Ayats J, Sendra M, Peña C (1999). Environmental sampling of Acinetobacter baumannii: moistened swabs versus moistened sterile gauze pads. Infect Control Hosp Epidemiol.

[25] Garner JS, Jarvis WR, Emori TG, Horan TC, Hughes JM (1988). CDC definitions for nosocomial infections, 1988. Am J Inf Control.

[26] Kennedy P. Forecasting with dynamic regression models: Alan Pankratz, 1991, (John Wiley and Sons, New York), ISBN 0-471-61528-5, [UK pound]47.50. Int J Forecast. Elsevier; 1992;8:647–8.

[27] Time Series Analysis and Forecasting - Second Edition [Internet]. [cited 2020 Sep 24]. http://www.scausa.com/toc.php

[28] Bracco D, Dubois M-J, Bouali R, Eggimann P (2007). Single rooms may help to prevent nosocomial bloodstream infection and cross-transmission of methicillin-resistant Staphylococcus aureus in intensive care units. Intensive Care Med.

[29] Mollers M, Lutgens SP, Schoffelen AF, Schneeberger PM, Suijkerbuijk AWM (2017). Cost of Nosocomial Outbreak Caused by NDM-1-Containing Klebsiella pneumoniae in the Netherlands, October 2015-January 2016. Emerging Infect Dis.

[30] Ng DHL, Marimuthu K, Lee JJ, Khong WX, Ng OT, Zhang W (2018). Environmental colonization and onward clonal transmission of carbapenem-resistant Acinetobacter baumannii (CRAB) in a medical intensive care unit: the case for environmental hygiene. Antimicrob Resist Infect Control.

[31] CDC and ICAN. Best Practices for Environmental Cleaning in Healthcare Facilities in Resource-Limited Settings. Atlanta, GA: US Department of Health and Human Services, CDC; Cape Town, South Africa: Infection Control Africa Network; 2019. https://www.cdc.gov/hai/prevent/resource-limited/index.html and http://www.icanetwork.co.za/icanguideline2019/

[32] Venditti C, Vulcano A, D’Arezzo S, Gruber CEM, Selleri M, Antonini M (2019). Epidemiological investigation of an Acinetobacter baumannii outbreak using core genome multilocus sequence typing. J Glob Antimicrob Resist.

[33] Bogaty C, Mataseje L, Gray A, Lefebvre B, Lévesque S, Mulvey M (2018). Investigation of a Carbapenemase-producing Acinetobacter baumannii outbreak using whole genome sequencing versus a standard epidemiologic investigation. Antimicrob Resist Infect Control.

[34] Dickstein Y, Lellouche J, Ben Dalak Amar M, Schwartz D, Nutman A, Daitch V (2019). Treatment Outcomes of Colistin- and Carbapenem-resistant Acinetobacter baumannii Infections: An Exploratory Subgroup Analysis of a Randomized Clinical Trial. Clin Infect Dis.

[35] Jones CL, Clancy M, Honnold C, Singh S, Snesrud E, Onmus-Leone F (2015). Fatal outbreak of an emerging clone of extensively drug-resistant Acinetobacter baumannii with enhanced virulence. Clin Infect Dis..

[36] Karakonstantis S, Gikas A, Astrinaki E, Kritsotakis EI (2020). Excess mortality due to pandrug-resistant Acinetobacter baumannii infections in hospitalized patients. J Hosp Inf.

[37] Bonomo RA (2019). Cefiderocol: a novel siderophore cephalosporin defeating carbapenem-resistant pathogens. Clin Infect Dis.

