# Supplementary material for: A five-component infection control bundle to permanently eliminate a carbapenem-resistant Acinetobacter baumannii spreading in an intensive care unit
Source: Antimicrob Resist Infect Control. 2021 Aug 19;10:123. doi: 10.1186/s13756-021-00990-z (PMC8376111; doi:10.1186/s13756-021-00990-z)
Supplement: Supplementary file 1 — Additional file 1. The comparison of different infection control interventions reported in literature for the management of CRAB outbreaks in ICUs. [file 13756_2021_990_MOESM1_ESM.pdf]

Study	Interventions													Outcomes		
	HH Compliance/AHR Consumption	Active Rectal Screening (Targeted/ Universal)	Additional Active Screening Strategies	Contact Isolation /Alert Code	Daily Chlorhexidine Baths	Cohorting Staff/patients	Closure/Stop Admissions	Environmental Disinfection	Environmental Cultures	Monitoring of Environmental Cleaning	Genotyping	Antimicrobial Stewardship/Monitoring of Antibiotic Consumption	Training /Education	Statistical method	Slope change	Linear change
Munoz-Price et al. (12) 2014	Hand imprints were episodically obtained from staff	No	Active surveillance through respiratory samples	Contact precautions + electronic communication	*NA*	Yes	*NA*	Bleach wipes (1:10 bleach)	Yes, weekly (39% positive)	Yes, using an ultraviolet monitoring system	PGFE	*NA*	Yes, with periodical reminders	SR: CRAB hospital acquired cases decreased	SC NS (0.07)	*NA*
Enfield et al. (13) 2014	*NA*	No	Wound and respiratory samples of all at-risk patients in ICU	Pre-emptive isolation for all patients	Yes	Yes	*NA*	Sodium hypochlorite	*NA*	Yes, using a specific checklist	PFGE	Yes, with direct audit	Yes, staff education, with audits and feedback in multi disciplinary meetings	ITS: CRAB_ID decreased	SC −4.81 (−7.00, −2.61)	LC −48.86 (−67.18, −30.54)
Gray et al. (14) 2016	*NA*	Yes, targeted on roommate only	Groin, throat, nose, wounds, and insertion sites	Contact precautions + elettronic flag on readmissions	Yes	Yes	*NA*	*NA*	*NA*	No	PFGE	No	Yes	*NA*		
Karampatakis et al. (15) 2018	AHR repositioning/ HH before aseptic procedures improved from 28.6% to 62.5% for doctors (p= 0.027) and from 50.0% to 75.9% for nurses (p = 0.034)	Yes, universal on admission and weekly	*NA*	Contact precautions and isolation of CRAB colonized patients /compliance improved from 46.1% to 66.6% for doctors (p= 0.009) and from 55% to 71% for nurses (p = 0.001)	*NA*	Yes	No	Sodium hypochlorite + Quaternary ammonium for sensitive electronic equipment	Yes, randomly taken with moistened swab (all negative)	*NA*	No	Yes, with direct audit three times Restricted formulary for carbapenems)/ non-significant increase of carbapenem (from 575.3 to 700.0 ddds per 1000 bed-days)	Yes, staff education, with audits and feedback in multi disciplinary meetings	ITS	linear trend of CMA for CRAB infections increased from 5.2 to 15.3 infections per 1000 patient-days (p = 0.001)	

Valencia-Martìn et al. (16) 2019	HH compliance monitored once each month and weekly reports: from 41.8 to 82.3% in the post-intervention (p < 0.001)	Yes, universal on admission and weekly	Throat weekly	Contact precautions and isolation of CRAB colonized patients /compliance improved from from 0 to 100%	*NA*	*NA*	Yes, sequentially closure and periodically at least thrice a year	Sodium hypochlorite + Benzalkonium chloride and propane-1,2-diol for sensitive electronic equipment	No	Yes, using a specific checklist	PFGE	Yes, with direct audit and feedback weekly: Antibiotic consumption decreased from 165.35 to 150.44 DDD/1000 patients-days,	Yes, staff education and feedback of the results	JP + ITS	SC -2,77% (-4.91, 0.57)	*NA*
Cho et al. (17) 2014	No data of HH compliance.AHR increased from 5.6 L to 119 L per 1000 patient-days	No	Nasal swabs of all ICU patients on admission and weekly	Contact precautions and isolation	No	Yes	*NA*	Sodium dichloroiso-cyanurate (nadcc)-containing solution	No	No	No	consumption of antimicrobials: total antimicrobials (R= 0.722; p < .001) and carbapenems (R = 0.530; p = 0 .008)	Yes, biweekly onsite	SR: CRAB_ID decreased	SC -0.013 (-0.02, -0.03)	LC 0.340 (0.14, 0.54)
Chung et al. (18) 2015	No change in direct compliance rates	Yes, universal	Nasal swabs	Contact precautions and isolation + Preemptive isolation	Yes	Yes	*NA*	Twice-daily cleaning of high-touch areas	Yes, swab system with selective chromogenic medium (chromagar acinetobacter) *1	Yes, using A specific checklist (from 30 to 6% were positive)	No	*NA*	Yes, on site	ITS: CRAB_ID decreased	SC -0.413 (-0.585, -0.240)	LC –0.066 (-0.309, 0.177)
Zhao et al. (19 2019	AHR repositioning	No	No	Contact precautions	*NA*	Yes	*NA*	Sodium hypochlorite	No	No	ERIC-PCR	Yes, restricted formulary for carbapenems and colistin	*NA*	CS	Prevalence of CRAB decreased from 87% to 11%	
Molter et al. (20) 2016	No data on HH compliance / AHR additional dispensers	Yes, universal on admission	Tracheal aspirates, throat, insertion sites, wounds, groins, on admission and weekly + Staff members screening	Contact precautions and isolation of CRAB colonized patients	*NA*	Yes	Yes, temporary stop of admissions	Glucoprotamine with single use tissue wipes	Yes standard method (all negative)	No	PFGE	No	Yes, weekly educational sessions for all personnel	*NA*		

Ben-chetrit et al. (21) 2018	Yes, significant increase in AHR and in HH compliance: from 84% to 97% in the post-intervention (p < 0.001)	Yes, universal on admission and weekly	Only sputum cultures twice per week	Contact precautions and isolation of CRAB colonized patients + thick red lines painted On the floor, "virtual walls"	only chlorhexidine-based soap available at sinks	Yes	Yes, unit closure (3 days) + evacuation of the "infected" ICU with transfer to A temporary ICU area	Sodium hypochlorite + Quaternary ammonium for sensitive electronic equipment	Yes, with a sterile gauze wetted with 5 ml of a selective mueller-hinton broth containing vancomycin (6 G/L) and ertapenem (2 G/L), 37% positive	Yes, adenosine triphosphate (atp) detection *2	WGS of CRAB blood isolates using a miseq benchtop sequencer *3	Yes, ICU meropenem, piperacillin-tazobcatam and colistin consumptions: only a significant decrease in use of colistin from 200 to 75 defined daily dose (DDD)/1000 patient-days (p < 0.05)	Yes, continuous education of staff with feedback and verbal audit	SR: CRAB_ID decreased	SC − 0.081 (− 0.131, − 0.031)
Metan et al. (22) 2019	No change in direct compliance rates: 59% vs 62% (p= 0.380)	Yes, target, weekly for the first month then monthly	Urine, throat, groin and axilla weekly for the first month then monthly	Contact precautions and isolation of CRAB colonized patients	Yes	Yes	Yes, ICU was closed for new admission for one month	Sodium hypochlorite	*NA*	*NA*	PFGE	*NA*	*NA*	BA: CRAB_ID decreased	from 3.186 to 2.820 cases per 1000 patient-days
Meschiari et al. 2020	ICU AHR increased From 30 L to 100 L per 1000 patient-days/ HH compliance was monitored periodically and results were included in weekly meeting: from 45% to 73% in the post-intervention (p < 0.001)	Yes on admission and weekly	Throat, groin and axilla on admission and weekly (for all patients staying more than 24 hours)	Contact precautions for all patients until discharge Isolations in single room for positive patients/ monitoring of compliance with a contact precaution specific checklists; Alert code with informatic flag	Yes	No	No	Daily 10% sodium hypochlorite solution for all environmental surfaces and hydrogen peroxide wipes for all medical devices/ periodically cycling disinfection	Yes, with moistened sterile gauze pads in a screw-cap container with 10 ml of brain heart infusion (BHI) an rubbing the surfaces to be sampled	Yes, using fluorescein spray and a check-list	WGS of clinical and environmental samples	Yes, with direct audit and feedback by an infectious disease specialist three times a week (restricted formulary) The total antibiotic consumption decreased from 209 to 171 DDD/1000 patients-days, carbapenems from 60 to 18 DDD/1000 patients-days	Yes, "improvement group" with all the ICU staff to analyse critical issues regarding infection control procedures;	ITSA: CRAB_ID decreased	significant decrease of - 2.9 (+/- 2*0.69) new nosocomial ICU-CRAB cases per 1000 patient-day
**All studies**	**7**/12 (58%): •**3**/12 (25%): increased AHR use •**4**/12(33%): increase in HH compliance	**8**/12 (67%): •**6**/8 (75%) universal •**2**/8 (25%) target	•**9**/12 (75%) respiratory sites •**4**/12 (33%) skin •**1**/12 (8%) staff	•**12**/12 (100%) contact precautions •**2**/12 (20%) alert code •**1**/12 (8%) pre-emptive isolation	**5**/12 (42%)	**10**/12 Yes (83%) **1**/12 No (8%) **1**/12 NA (8%)	**4**/12 Yes (33% **2**/12 No (16%) **6**/12 NA (50%	**11**/12 (92%): •**10**/11 (90%) sodium hypochlorite •**1**/11 (9%) cycling disinfection	**6**/12 (50%) : •**3**/6 (50%) with selective medium (2 with moistened gauze)	**6**/12 (50%) : •**3**/6 (50%) check-list •**1**/6 uv •**1**/6 atp •**1**/6 fluorescent marker	**9**/12 (75%): •**6**/9 (67%) PFGE •**2**/9 (22%) WGS •**1**/9 (11%) ERIC-PCR	**6**/12 (50%): •**3**/6 (50%) carbapenems	**10**/12 (83%)	**9**/12 (75%) : favourable outcome	

Supplementary Table 1. Comparison of different infection control interventions reported in literature for managing carbapenem-resistant *Acinetobacter baumannii* (CRAB) outbreaks in intensive care units (ICU).

Legenda: AHR alcohol-based hand rubs; BA Before and after study; BHI brain heart infusion medium; CMA centred moving average; CRAB carbapenem-resistant Acinetobaster baumannii; CRAB_ID CRAB incidence density; CS cross-sectional study; DDD Defined Daily Doses; ERIC-PCR Enterobacterial repetitive intergenic consensus; HH hand hygiene; ICU intensive care unit; ITS interrupted time-series analysis; ITSA Intervention time series analysis ; JP joinpoint regression analysis; LC linear change; NA not available; PGFE Pulsed-field gel electrophoresis; SC slope change; SR segmented regression analysis; BA before and after analysis; WGS whole-genome sequencing.

*1 3M quick swabs; 3M, St Paul, MA; *2 Atp complete® contamination monitoring system; ruhof, mineola, NY, USA; *3 Illumina inc., San Diego, CA, USA.
