# Supplementary material for: A five-component infection control bundle to permanently eliminate a carbapenem-resistant Acinetobacter baumannii spreading in an intensive care unit
Source: Antimicrob Resist Infect Control. 2021 Aug 19;10:123. doi: 10.1186/s13756-021-00990-z (PMC8376111; doi:10.1186/s13756-021-00990-z)
Supplement: Supplementary file 2 — Additional file 2. Microbiological analysis and whole genome sequencing analysis are illustrated technically. Patients’ history, clinical course and characteristics are fully described in text and in Supplementary Table 2. Antimicrobial susceptibility testing of the CRAB clinical isolates is shown in Supplementary Table 3. [file 13756_2021_990_MOESM2_ESM.docx]

# **A novel infection control bundle to permanently eliminate a carbapenem-resistant *Acinetobacter baumannii* spreading in an intensive care unit.**

**SUPPLEMENTARY MATERIAL**

The Supplementary Material includes both text and tables.

**Microbiological Analysis**

All collected *Acinetobacter baumannii* isolates were identified by MALDI-TOF MS using VITEK MS (bioMérieux, Marcy l´Etoile, France) following the manufacturer’s instructions. Antimicrobial susceptibility testing was performed by microdilution method using a testing panel MICRONAUT Merlin, Germany). Susceptibility testing for cefiderocol was carried out using iron-depleted cation-adjusted Mueller-Hinton broth (ID-CAMHB) and frozen broth microdilution panels provided by IHMA (International Health Management Associates, Inc., US). *P. aeruginosa* ATCC 27853 was used as QC strain. MICs were interpreted according to the EUCAST breakpoints, Version 10.10, 2020 (25) (and addendum Cefiderocol breakpoints and AST methods).

**Whole Genome Sequencing Analysis**

Clinical isolates for genotyping were selected giving priority to diagnostic samples from sterile body sites, surveillance samples were included only in the absence of clinical ones in order to collect at least one sample per patient.

Total bacterial DNA was extracted from pure cultures using the Qiagen DNeasy PowerLyzer PowerSoil Kit (QIAGEN). All DNA solutions were stored at -20°C until further use. Purified genomic DNA samples were used to prepare shotgun libraries which were then subjected to whole-genome sequencing with the Illumina HiSeq platform (Illumina, Inc., San Diego, CA), using a 2x150 bp paired-end approach. Sequence reads were assembled using SPAdes v3.10.0 (Bankevich et al. 2012). Genome annotation was performed using the NCBI Prokaryotic Genome Annotation Pipeline (PGAP) (https://www.ncbi.nlm.nih.gov/genome/annotation_prok/). *In silico* analyses were performed using dedicated tools available at the Center for Genomic Epidemiology (https://cge.cbs.dtu.dk/). Clonal relatedness was investigated by *in silico* determination of Multi-Locus Sequence Typing (MLST) profiles obtained using MLST 2.0, and by generation of core-genome single nucleotide polymorphisms (SNPs)-based phylogenetic trees. To infer the global phylogeny including all sequenced isolates, core genome alignments were generated using Roary (26) and variable positions were extracted from the alignment and used to generate a maximum likelihood tree with IQ-TREE v1.6.12, using a general time reversible model (27). Raw sequencing data and draft genome assemblies of *A. baumannii* strains reported in this study have been deposited at NCBI under the BioProject accession number PRJNA625184.

**Patient history and clinical course**

The index patient (P1) came from Greece and was admitted to the ICU on February 25^th^ while waiting for a liver retransplant after receiving a first transplant in Athens in January 2018. CRAB was isolated from the surveillance rectal swab taken on admission. The following day P1 developed a CRAB ventilator-associated pneumonia and on February 27^th^ a CRAB bloodstream infection (BSI). The day after the detection of rectal colonization, P1 was moved to a closed isolation room and another liver transplant patient (P2) was moved from the isolation room to the empty area next to the area where P1 previously was. P2, a 58-year-old woman, died within 24 hours of CRAB BSI and septic shock.

P1’s BSI was controlled, but the patient remained colonized by CRAB and developed subsequent recurrent CRAB infections. At the end of March, his condition deteriorated. The patient eventually developed a new BSI episode by CRAB and died on April 12^th^.

During March, five additional patients in the ICU were found positive for CRAB.

P3 was a 9-year-old child admitted from Emergency department to ICU for a traffic polytrauma with massive facial breakage, hemoperitoneum and intracranial hemorrhage. Skin and throat samples collected four days after admission were positive for CRAB. On March 13^rd^ P3 was transferred to Pediatric department. P4, a 52-year-old oncologic patient with metastatic ovary cancer, was admitted to ICU after undergoing peritonectomy and hyperthermic intraperitoneal chemotherapy. Urinary CRAB colonization was found ten days after admission. P4 was discharged from ICU on March 23^rd^.

P5, a 73-year-old man, with an history of pharyngeal and laryngeal cancer previously treated with radiation therapy and radical surgery, was admitted to ICU on February 27^th^ with severe respiratory insufficiency due to a hospital-acquired pneumonia (HAP) that required orotracheal intubation. On March 15^th^ bronchoalveolar-lavage yielded CRAB and on March 27^th^ he died from respiratory failure. P6, a 66-year-old dialyzed diabetic woman with osteomyelitis, was admitted on March 11^st^ from dialysis center for septic complications following lower limb amputation. On March 22^nd^ she developed a septic shock with CRAB isolation from blood, bronchial aspirate, surgical wound, urine and skin samples and died from multi-organ failure two days later. P7 was an 83-year-old woman affected by a high-grade lymphoma with a history of long hospitalization in hematology ward. On March 26^th^ P7 was admitted to ICU for peritonitis secondary to a bowel perforation, three days later she developed a CRAB BSI and died with septic complications.

CRAB positivity appeared an average of 14 ±12.1 days after hospitalization and of 7 ± 5.8 days after ICU admission. The prevalent site of colonization was skin and respiratory tract (both 100%), followed by the rectum (85.7%). The most frequent site of CRAB infection was bloodstream infection (4 patients) followed by ventilator associated pneumoniae (2 patients).

Antimicrobial susceptibility testing of the CRAB clinical isolates from the seven patients (two isolates from different sources were available for P1 and P3) showed uniform phenotypic resistance to carbapenems (imipenem and meropenem), aminoglycosides (gentamicin and amikacin), trimethoprim-sulfamethoxazole, and ciprofloxacin, while most isolates (except those from P6 and P7) were also resistant to colistin. On the other hand, all isolates were susceptible to cefiderocol and showed relatively low MIC values for tigecycline (**Supplementary Table 3**).

Concerning treatment, P1 and P5 were treated with cefiderocol (40) via the Italian compassionate use approval procedure. P2, P6 and P7 were treated with various combination therapies based on synergistic tests, including carbapenems, tigecycline, colistin, rifampicin, and ampicillin-sulbactam. All infected patients died from CRAB septic shock. P3 and P4, who were only colonized, survived. In-hospital mortality was 71.4%. Of these, 77% were attributable to CRAB sepsis.

**Supplementary Table 2. Demographic and clinical features of patients with carbapenem-resistant *Acinetobacter baumannii* (CRAB) infection or colonization, February-March 2018.**

| **Variable** | **Mean (±DS) or N (%)** |
| --- | --- |
| Number of patients | 7 |
| Age (years) | 57 (±23) |
| Male sex | 1 (14.3) |
| Pattern of acquisition  Infection  Colonization | 5 (71.5)  2 (28.5) |
| Outcome  Death during hospitalisation  Death attributable to CRAB sepsis | 5 (71.4)  3 (42.8) |
| Risk factors  Diabetes mellitus  Chronic obstructive pulmonary disease  Previous hospitalization  Chronic kidney disease  Cancer  Trauma  Steroid therapy  Immunosuppressants  End-stage liver disease  Cardiovascular disease | 0  0  4 (57.1)  1 (14.3)  3 (42.9)  1 (14.3)  3 (42.9)  4 (57.1)  2 (28.6)  0 |
| Multimorbidity (>=2) | 5 (71.4) |
| Diagnosis at admission  Coronary artery disease  Liver failure  Pneumonia  Septic shock  Cancer  Respiratory failure  Kidney failure  Trauma  Post surgery | 0  2 (28.6)  1 (14.3)  3 (42.9)  0  3 (42.9)  2 (28.6)  2 (28.6)  1 (14.3) |
| Device  Central venous catheter  Endotracheal tube  Drainage tube | 6 (85.7%)  6 (85.7%)  3 (42.9%) |
| SOFA score | 8.7 (±7) |
| CRAB-positive samples collected for body site  Rectal  Skin  Bronchoalveolar lavage  Endotracheal aspirate  Blood  Central venous catheter  CNS  Urine | 6 (85.7)  7 (100)  4 (57.1)  2 (28.6)  4 (57.1)  4 (57.1)  0 (0)  3 (42.9) |
| Type of infection  Total  Intrabdominal  Bloodstream infection  Pneumonia  Skin and soft tissue | 5  0  4  2  0 |
| ICU admission (days of hospitalization) | 9 (±9.7) |
| Discharge/death (days of hospitalization) | 26 (±18.5) |
| Discharge/death (days in ICU) | 19 (±17.9) |
| ICU stay before colonization (days) | 7 (±5.8) |
| Hospital stay before colonization (days) | 14 (±12.1) |
| ICU stay before infection (days) | 7 (±6.3) |
| Hospital stay before infection (days) | 17 (±13.6) |
| Time from colonization to infection (days) | 0 (±0.1) |

**Supplementary Table 3. Minimal inhibitory concentration (MIC) values of the carbapenem-resistant *A. baumannii* (CRAB) isolates.**

| **Patient number** | **Isolate number** | **GEN** | **AMK** | **IPM** | **MEM** | **CIP** | **TZP** | **SXT** | **SAM** | **CST** | **TGC** | **FDC** |
| --- | --- | --- | --- | --- | --- | --- | --- | --- | --- | --- | --- | --- |
| P1 | A1.1 | >4 | >16 | >16 | 128 | >2 | >128 | >4 | >32 | 8 | 2 | 1 |
|  | A1.2 | >4 | >16 | >16 | 128 | >2 | >128 | >4 | >32 | >8 | 2 | 1 |
| P2 | A2.1 | >4 | >16 | >16 | 128 | >2 | >128 | >4 | >32 | >8 | 2 | 2 |
| P3 | A3.1 | >4 | >16 | >16 | 64 | >2 | >128 | >4 | >32 | >8 | 1 | 1 |
|  | A3.2 | >4 | >16 | >16 | 64 | >2 | >128 | >4 | >32 | >8 | 1 | 1 |
| P4 | A4.1 | >4 | >16 | >16 | 64 | >2 | >128 | >4 | >32 | >8 | 1 | 1 |
| P5 | A5.1 | >4 | >16 | >16 | 64 | >2 | >128 | >4 | >32 | >8 | 1 | 1 |
| P6 | A6.1 | >16 | >16 | >16 | >64 | >2 | >128 | >320 | >32 | <=1 | 1 | 0.5 |
| P7 | A7.1 | >16 | >16 | >16 | >64 | >2 | >128 | >320 | >32 | <=1 | 1 | 0.5 |

Isolates were tested with the MICRONAUT S-MDR panel (Merlin, Germany).

amikacin (AMK); ampicillin-sulbactam (SAM); ciprofloxacin (CIP); colistin (CST); gentamicin (GEN); imipenem (IPM); meropenem (MEM); piperacillin-tazobactam (TZP); tigecycline (TGC); trimethoprim-sulfamethoxazole (SXT); cefiderocol (FDC) from <https://aac.asm.org/content/abbreviations-and-conventions>.
